# Structural insights of anti-MHC-I monoclonal antibodies that block NK cell receptor interactions with tumors

**DOI:** 10.1063/4.0001114

**Published:** 2025-10-27

**Authors:** Jiansheng Jiang, Kannan Natarajan, Abir Panda, Lisa F. Boyd, Reanne Towler, Haotian Lei, Rick Huang, Ethan M. Shevach, David H. Margulies

**Affiliations:** 1 NIAID/NIH, Bethesda, Maryland, USA; 2 CCR/NCI, Bethesda, Maryland, USA

## Abstract

We have recently shown that certain anti-MHC-I mAbs in human and murine systems can stimulate immune responses to tumors and infections by competitively blocking inhibitory signals delivered by MHC-I-specific inhibitory receptors of several inflammatory cell types **[1,2]**. To understand such functions, we determined the structure of a broadly reactive anti-human MHC-I mAb, B1.23.2, in complex with the MHC-I molecule HLA- B*44:05 by both X-ray crystallography and cryo-electron microscopy (cryo-EM). Structural models determined by the two methods were identical and revealed that B1.23.2 binds a highly conserved site on the a2-1 helix. Structural comparison to killer immunoglobulin receptors (KIR)/MHC-I (HLA) complexes reveals a mechanism by which B1.23.2 blocks inhibitory KIR interactions, leading to natural killer (NK) cell activation (Figure.1). The function of such anti-MHC-I mAbs to block the interactions with NK inhibitory receptors is distinct from most available mAbs that bind the NK cell receptor directly. Applying such anti-MHC-I mAbs to suppress tumor growth may offer new avenues for immunotherapeutic intervention against cancer.
